# Join the club: ORP8 is a lipophagy receptor

**DOI:** 10.1093/procel/pwad005

**Published:** 2023-02-09

**Authors:** Zheng Wang, Hong Zhang

**Affiliations:** National Laboratory of Biomacromolecules, Institute of Biophysics, Chinese Academy of Sciences, Beijing 100101, China; National Laboratory of Biomacromolecules, Institute of Biophysics, Chinese Academy of Sciences, Beijing 100101, China; College of Life Sciences, University of Chinese Academy of Sciences, Beijing 100049, China

Autophagy involves engulfment of cytosolic constituents in a *de novo*-synthesized double-membrane autophagosome and its subsequent delivery to the lysosome for degradation and recycling of sequestrated materials (Nakatogawa, 2020; Zhao et al., 2021). In terms of the cargo enclosed by the autophagosome, autophagy can be nonselective, in which a portion of the cytosol is indiscriminately sequestered; or highly selective, in which specific cargoes such as protein aggregates, superfluous/damaged organelles, or invading pathogens are engulfed (Vargas et al., 2022). Selective autophagy can be classified into different types according to the cargo, such as aggrephagy (protein aggregates), ER-phagy (endoplasmic reticulum), mitophagy (mitochondria) and lysophagy (lysosomes) (Vargas et al., 2022).

**Figure 1.**
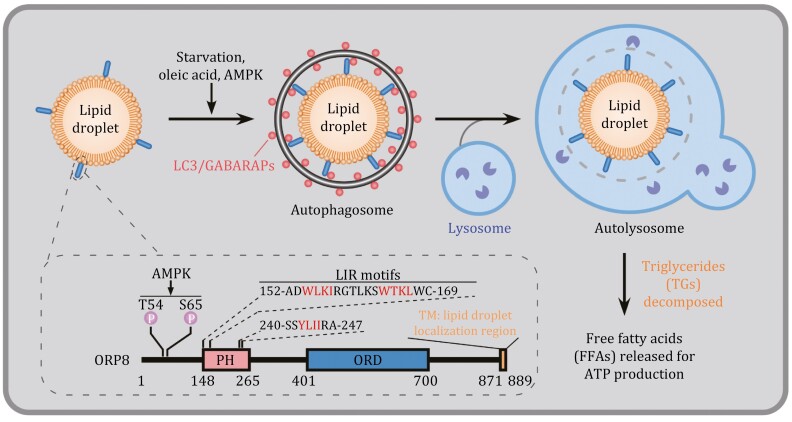
**ORP8 acts as the receptor for autophagic turnover of lipid droplets.** ORP8 is localized on the lipid droplets (LDs) via its C-terminal transmembrane (TM) domain. Upon lipophagy induction, for example, by oleic acid (OA) treatment or starvation, ORP8 on LDs interacts with LC3/GABARAPs, thus acting as a receptor to mediate the delivery of LDs to the lysosome for degradation to release free fatty acids (FFAs). Three LIR motifs within the PH domain mediate the binding of ORP8 to LC3/GABARAPs upon lipophagy induction. This binding is facilitated by AMPK-mediated phosphorylation at the Thr54 and Ser65 sites of ORP8.

During selective autophagy, a family of receptor proteins render the cargo capable of engaging with the autophagosomal precursor, called the isolation membrane (IM), with tight juxtaposition (Sawa-Makarska et al., 2014). The receptor recognizes cargoes via direct interaction, or indirectly such as via binding to a tag (e.g., polyubiquitin chains) added onto the cargo (Vargas et al., 2022). The receptor also interacts with LC3/GABARAP family autophagy proteins, which are conjugated to phosphatidylethanolamine on the IM (Johansen et al., 2020). The receptor can also recruit upstream autophagy proteins to initiate the formation of IMs surrounding cargoes (Vargas et al., 2022). Distinct receptors are utilized for degrading different cargoes. For example, p62, NBR1, TAXIBP1 and SEPA-1 mediate degradation of different protein cargoes; FAM134B, SEC62 and TEX264 act in ER-phagy; while FUNDC1, NDP52 and OPTN function in mitophagy (Chino and Mizushima, 2023; Vargas et al., 2022). Extensive studies have shown that the lipid droplet (LD), an organelle filled with neutral lipids such as triglycerides (TGs) and surrounded by a phospholipid monolayer, can be selectively degraded by autophagy, a process known as lipophagy. The receptor mediating this process, however, remains elusive. The gap is now filled by a study from the Liu lab published in this issue, showing that ORP8, a member of the oxysterol binding protein (OSBP) family, acts as a receptor to mediate lipophagy (Fig. 1) (Pu et al., 2022).

LD biogenesis and turnover are tightly controlled to maintain lipid and energy homeostasis in cells (Zechner et al., 2017). To uncover potential receptors mediating lipophagy, Pu et al. performed mass spectrometry analysis to identify LC3-associated LD proteins. LDs purified from cells treated with oleic acid (OA) (to stimulate LD biogenesis) and chloroquine (CQ) (to block lysosomal degradation) were lysed and affinity-precipitated by GST-LC3B. ORP8 was identified from the precipitates. ORP8, but not other OSBP family members such as OSBP, ORP2 and ORP5, interacted with LC3 upon lipophagy induction. ORP8 is an ER-resident lipid transporter protein mediating the counter-transport of phosphatidylinositol 4-phosphate (PI4P)/phosphatidylserine (PS) between the ER and the plasma membrane (Chung et al., 2015). The authors found that ORP8 is also targeted to LDs via its C-terminal transmembrane (TM) domain, while the N-terminal Pleckstrin homology (PH) domain and the lipid transfer ORD domain are dispensable. Distribution of ORP8 to LDs was enhanced upon lipophagy induction.

The authors then examined LD catabolism in *ORP8*-depleted or -overexpressing cells. LD content was dramatically increased by *OPR8* knockout (KO) and significantly reduced by OPR8 overexpression. In cells with elevated LD biogenesis or consumption, ORP8 also modulated LD catabolism. Upon blocking LD biogenesis with the fatty acyl-CoA synthetase (ACS) inhibitor triacsin C, LD turnover was promoted in control but not in *ORP8* KO cells, while in cells depleted of adipose triglyceride lipase (ATGL), simultaneous knockout of ORP8 further increased the LD content (Pu et al., 2022). The lipid transporter activity of ORP8 is not required for its function in LD turnover, as expression of the lipid transport-inactive ORP8-H514A/H515A mutant still reduced the LD content (Pu et al., 2022). Pu et al. then provided evidence to show that ORP8 regulates LD turnover via lipophagy. First, *ORP8* KD failed to further increase the LDs in cells depleted of the essential autophagy gene *ATG7*. Second, *ORP8* KO cells contained fewer red-only puncta (mCherry^+^ GFP^−^), detected by using the mCherry-GFP-liverdrop LD marker. Formation of red puncta indicates the delivery of LDs into lysosomes due to quenching of the acidity-sensitive GFP signal. Thus, less LDs were delivered into lysosomes in *ORP8* KO cells. Third, co-localization of LDs with LC3 was decreased and lipid-containing autophagosomes were fewer in *ORP8* KO cells than control cells under starvation conditions. *ORP8* KO did not affect degradation of protein substrates such as p62, or autophagosome formation. Thus, ORP8 promotes LD turnover via lipophagy.

The LD localization and facilitation of lysosomal LD turnover raised the possibility that ORP8 acts as a lipophagy receptor. An essential property of an autophagic receptor is its binding to LC3 family members. Pu et al. found that ORP8 interacts with LC3-labeled autophagic structures. LDs purified from control but not *ORP8* KO cells were recruited by GFP-TRAP beads that were trapped with GFP-LC3-positive membranes. Co-immunoprecipitation analysis revealed the interaction between endogenous ORP8 and lipidated LC3 (LC3-II), and this interaction was enhanced by induction of lipophagy. ORP8 also directly bound to LC3/GABARAP family proteins. Three LC3-interacting regions (known as LIR motifs) in ORP8, amino acids 154–157, 164–167 and 242–245, were delineated to mediate the interaction between ORP8 and LC3/GABARAPs (Fig. 1). Mutating the first motif (W154A/I157A), or the second motif (W164A/L167A), or all three LIR motifs (LIRs-6A) dramatically reduced ORP8 binding to LC3/GABARAP. The ORP8-LIRs-6A mutant failed to co-localize with LDs, or rescue the lipophagy defect in *ORP8*-deficient cells. As a receptor, ORP8 was also delivered to the lysosome for degradation.

Accumulation of cargoes stimulates the corresponding selective autophagy via multiple mechanisms such as posttranslational modification of receptors to enhance the binding affinity to the cargo or ATG8/LC3 family members. Pu et al. also addressed the mechanism which couples ORP8-mediated lipophagy with energy status. Lipophagy induction (e.g., by OA or starvation treatments) activated AMPK, which in turn bound to and phosphorylated ORP8. Mass spectrometry analysis identified Thr54 and Ser65 as phosphorylated sites in ORP8. Mutating Thr54 (T54A) or Thr54/Ser65 (T54A/S65A) greatly reduced, while mutating Ser65 (S65A) slightly reduced, the phosphorylation level of ORP8. The ORP8 (T54A/S65A) mutant still localized on LDs, but its binding to LC3/GABARAP was impaired, and it did not restore the delivery of LDs to lysosomes in *ORP8* KO cells. AMPK also modulates autophagy proteins such as the ULK1 initiating complex for IM formation. Therefore, AMPK-mediated phosphorylation of ORP8 integrates nutrient status into lipophagy.

The physiological function of ORP8-mediated lipophagy was examined in *ob*/*ob* mice and *Osbpl8* (encoding mouse ORP8) KO mice. Expression of ORP8, but not T54A/S65A or LIRs-6A mutant ORP8 in *ob*/*ob* mice significantly reduced the hepatic TG level and the accumulation of LDs in the liver. *Osbpl8* KO mice also exhibited significantly increased liver LDs and TG levels on a normal diet. Thus, ORP8-mediated lipophagy regulates LD catabolism in mouse models.

Altogether, this seminal study provides compelling evidence to show that ORP8 acts as a lipophagy receptor that can sense energy availability through AMPK-regulated interaction with LC3/GABARAPs. This study also raises many questions for future investigation. When and how does the ER-resident ORP8 translocate to LDs? ORP8 also functions with ORP5 at the mitochondria-associated membrane (MAM)-LD contact sites for LD biogenesis (Guyard et al., 2022). How are the dual functions of ORP8 in LD biogenesis and degradation balanced? Meanwhile, the discovery of ORP8 as a lipophagy receptor may be harnessed to develop strategies for treatment of lipid metabolic disorders.
